# Does extracellular vesicle specificity truly exist?

**DOI:** 10.1186/s12964-026-02654-0

**Published:** 2026-01-27

**Authors:** Subhashini Muhandiram, Alireza Fazeli

**Affiliations:** 1 Institute of Veterinary Medicine and Animal Sciences, Estonian University of Life Sciences, Tartu, Estonia; 2 Department of Pathophysiology, Institute of Biomedicine and Translational Medicine, University of Tartu, Tartu, Estonia; 3 Division of Clinical Medicine, School of Medicine and Population Health, The Medical School, University of Sheffield, Sheffield, UK

**Keywords:** Extracellular vesicles, Specificity, Tropism, Cell–cell communication

## Abstract

Extracellular vesicles (EVs) are nano-sized membranous particles secreted by nearly all cell types that facilitate intercellular communication through the transfer of bioactive cargo. Growing evidence suggests that EVs may exhibit targeting specificity toward particular cells, yet the mechanisms remain poorly understood. In this review, we critically examine the concept of EV target specificity by outlining three potential stages where it may arise: (1) EV binding to the recipient cell membrane, (2) internalization and cargo release and (3) the induction of functional responses within recipient cells. We further explore how EV-intrinsic properties, recipient cell characteristics, and the surrounding microenvironment collectively shape these interactions. A key challenge in the field is the frequent assumption that EV uptake equates to functional relevance. In reality, many internalized EVs are recycled or degraded in lysosomes without eliciting any measurable effect. We therefore propose a refined definition of EV specificity, emphasizing that functional outcomes should serve as the central criterion for establishing meaningful EV specificity. Rather than offering definitive answers, this review highlights unresolved questions and calls for deeper investigation into how EVs select their target cells. Understanding these fundamental principles of EV function is critical for advancing the clinical and therapeutic application of EVs.

## Introduction

Extracellular Vesicles (EVs) are nano-sized membranous particles secreted by nearly all cell types. They serve as crucial mediators of intercellular communication in physiological conditions and exploited for progression of pathological processes. These vesicles carry a variety of molecules from their donor cells and deliver this information to recipient cells, enabling them to decode and respond to the bioactive messages contained within. Through this mechanism, cells can exchange signals and influence each other's behaviors [1–4].

**Fig. 1:**
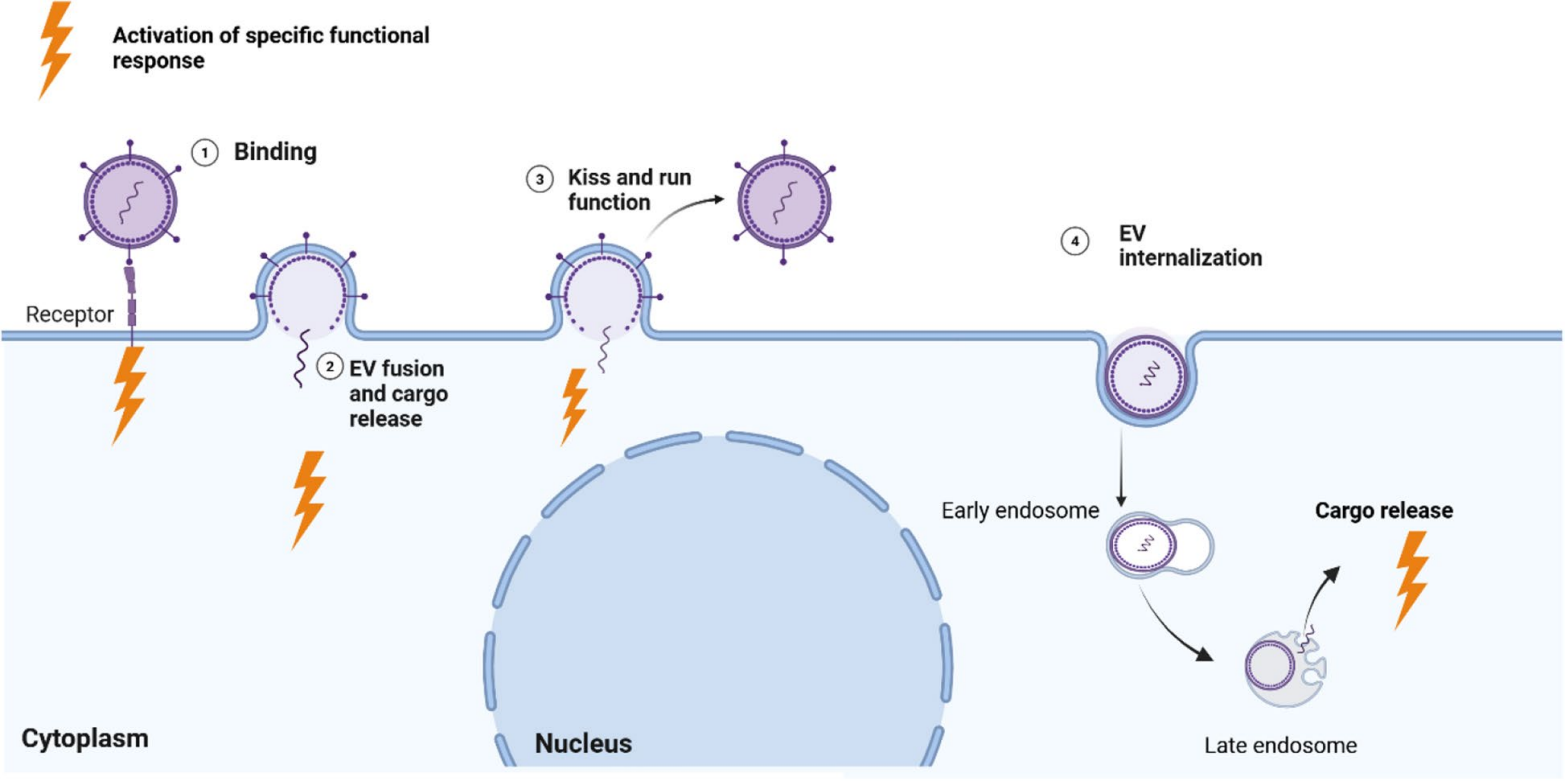
Multiple proposed mechanisms through which extracellular vesicles (EVs) may interact with target cells to induce a functional response. (1) EVs may interact with receptors on the recipient cell membrane, initiating downstream signalling pathways. (2) EVs can directly fuse with the recipient cell surface, releasing their cargo in to the cytosol and activating downstream signalling pathways. (3) EVs may partially fuse with the recipient cell membrane releasing part of their cargo while the remaining vesicle is recycled or re-released. (4) EVs can be internalized through various endocytic routes, followed by intracellular cargo delivery that trigger functional response

Effective communication between cells is vital for maintaining homeostasis in biological systems. In EV field, it is generally assumed that EVs released by one cell can influence any nearby or distant cell, regardless of type. Many EV functional studies have considered mechanism of action of EVs as "one-size-fits-all" model. Supporting this view, numerous uptake studies have shown that EVs can be internalized by a wide variety of cell types, suggesting that EV uptake may occur in a largely random or nonspecific manner [5, 6]. However, this assumption has gone largely unchallenged and warrants more rigorous investigation. Is it truly accurate to consider EV communication as a universal and equal mechanism among all cell types? Do EVs as mediators of intercellular communication has the ability to fine tune their signalling depending on the donor and recipient cell type, and if so, what factors influence this? For EVs to function as a reliable communication channel, they must target the right recipient cells at right time. Hence, it is understandable and even expected that certain EV types should show tropism/attraction to certain cell types. A growing body of evidence suggests that EVs may carry specific messages meant for selected cells [7–10]. However, much remains to be explored regarding this concept. It is already important to explain in this manuscript, what we mean by EV “specificity” and EV “tropism”. The term EV “specificity” refers to the selective delivery of EVs and their cargo to particular cell types or tissues, whereas EV “tropism” refers to the natural tendency of EVs to accumulate or interact with specific tissues, organs, or cell types**,** much like viral tropism. Both EV specificity and EV tropism essentially describe the same concept. If EV specificity exists, how do EVs ensure that the right message reaches the right cell at the right time? Answering this question requires a deeper understanding of the mechanisms through which EVs act on recipient cells, an area that remains only partially understood. While defining a universal mechanism for all EV types is challenging, several key checkpoints are known to regulate how EVs interact with recipient cells, such as EV binding or fusion at the cell surface, EV internalization, and cargo release before a functional response is initiated. Figure 1 illustrates multiple mechanisms through which EVs interact with target cells and ultimately elicit a functional response (Fig. 1) [11, 12].

If EVs are meant to induce specific functional responses, do these responses occur at distinct checkpoints along the interaction pathway? Is EV specificity determined at the level of selective binding to the recipient cell, or does it emerge only after EV internalization and cargo processing has taken place? Could it be that each stage of EV interaction—binding, internalization, and cargo release—triggers different types of responses, with some being highly specific and others more general? These questions highlight the complexity of EV-mediated communication and the need to better understand how functional outcomes are shaped throughout the interaction process.

Nevertheless, several potential scenarios can be envisioned for how EV specificity operates at different stages of EV-cell interaction. In the first scenario, EVs can convey specific messages simply by binding with the receptors on recipient cell membrane and inducing specific signalling pathways. In the second scenario, EV uptake itself is selective, occurring only in specific cell types. Here, cargo delivery and downstream responses are restricted to cells capable of internalizing the EVs, making uptake the key determinant of specificity. Thirdly, EVs bind to or are taken up by a broad range of cell types, but the specificity of the communication is governed by what happens after uptake, namely cargo release and the unique downstream responses of the recipient cell. Beyond the mechanisms described above, factors such as EV dose and exposure time play a crucial role in shaping their functionality and are essential for understanding their target specificity. However, studies examining standardized dose- and time-dependent responses to EVs are still limited. Therefore, it remains challenging to define EV functionality in terms of standardized dose–response and time-response frameworks [13, 14]. Evidence suggests that EVs can elicit distinct biological effects depending on the dose with varying thresholds triggering different functional outcomes [15]. Interestingly, a recent study showed specific EV responses are more pronounced with low EV dose [16]. Similarly, the timing of EV exposure plays a critical role, as some EVs provoke rapid, acute responses, while others induce more gradual or sustained effects [17, 18]**.** Yet, it is still unclear whether EV targeting specificity is more pronounced during acute interactions or under prolonged exposure, highlighting a key gap in our current understanding. On the other hand, EVs represent a heterogeneous population of particles, and it remains unclear to what extent their physicochemical properties influence their targeting specificity. Some studies suggest that factors such as EV size and surface charge can affect their uptake and functional impact on target cells [19, 20]. Moreover, the cellular microenvironment including local pH, receptor availability, and extracellular matrix composition may also modulate targeted intercellular communication [21, 22]. These nuanced yet critical factors are often overlooked, despite their importance in shaping our understanding of EV specificity.

Finally, after considering all these factors, we believe there is a need to revise the fundamentals of EV specificity. When do we truly call EVs specific? To answer this question, the most fundamental question we must address is: What exactly are EVs?

### What are EVs and what do they do?

**Fig. 2:**
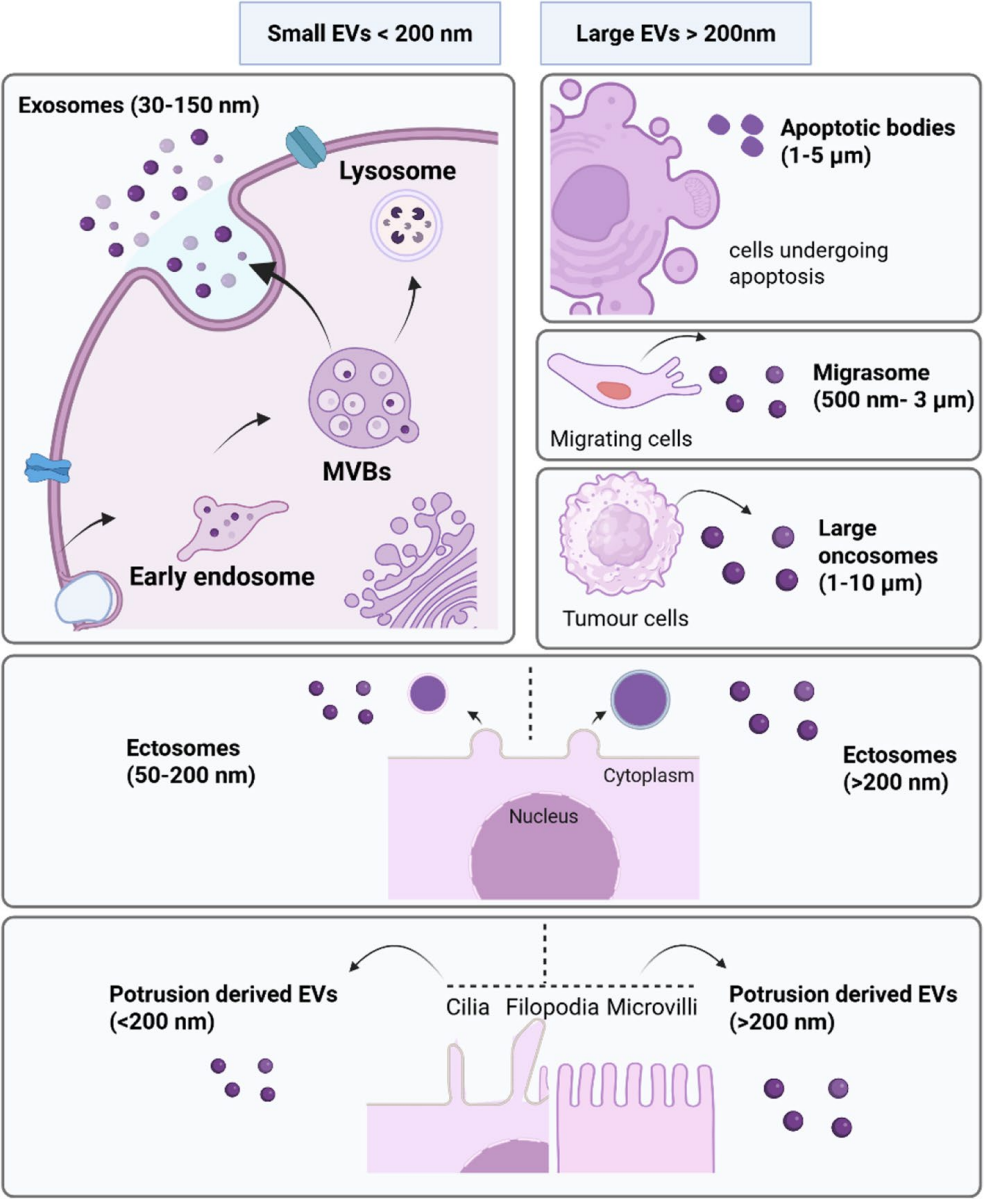
Schematic representation of heterogeneity of extracellular vesicles (EVs) and emerging EV subtypes. EVs are broadly classified into two main categories based on size: small EVs and large EVs. Beyond size, EVs can also be distinguished by their biogenesis pathways. Exosomes are EVs that originate from the endosomal system, specifically from multivesicular bodies (MVBs) and are typically < 200 nm in diameter [35]. EVs can also arise from specialized cellular processes—for example, apoptotic bodies during programmed cell death [36], migrasomes during cell migration [37], and large oncosomes released during tumorigenesis, and are usually > 200 nm in size [38]. Another major class includes EVs generated directly from the plasma membrane, commonly referred to as ectosomes [39]. In addition, cells can release EVs from dynamic membrane protrusions such as cilia, filopodia, and microvilli; these are known as protrusion-derived EVs (PDEVs) [40, 41]. Both ectosomes and PDEVs exhibit a broad range of sizes. MVBs; Multivesicular bodies

According to Minimal information for studies of extracellular vesicles (MISEV2023), EVs are defined as “particles that are released from cells, are delimited by a lipid bilayer, and cannot replicate on their own” [23]. EVs were described initially as performing a role in the cellular waste disposal [24] and recycling [25]. However, they are currently known to play vital functions, such as the maintenance of cellular homeostasis [26, 27] and intercellular signalling in different physiological and pathological conditions by carrying a range of bioactive cargoes including proteins, lipids and nucleic acids to neighbouring or distant recipient cells [27–29]. Interestingly, EVs have been isolated from almost all biological fluids, highlighting how widely they participate in biological processes [30–34]. Even though EVs can be categorized in to multiple subtypes based on biogenesis pathway, size and content (Fig. 2), it is now widely recognized that commonly used EV isolation techniques cannot reliably separate these subpopulations, and specific markers for each subtype remain limited. Therefore, the latest MISEV guidelines recommend using simpler and more practical operational terms such as “small EVs” and “large EVs” to describe EV populations released by cells.

The term “extracellular vesicles” is used as an umbrella term to represent all above vesicle types released by cells. It is remarkable to note that an average cell in its life time seems to be producing billions of different EVs [42] and large pool of evidence suggests that a portion of these vesicles is actively secreted for the purpose of intercellular communication. However, the question remains if these EVs are made to confer specific signals to specific cells or cell types in specific tissues or just target any cells at random?

### What is EV target specificity and why it is important?

#### Definition

EV target specificity refers to the idea that EVs are "programmed" to reach specific target cells once they are released into the extracellular environment. They are destined to find these cells within a complex multicellular system. Successful delivery of EVs to the right cell type at the right time ensures effective intercellular communication, which is essential for maintaining homeostasis in biological systems. According to the literature, EV specificity can occur at multiple levels, including EV binding, internalization, cargo delivery, and functional response, and distinguishing these stages and their underlying mechanisms is essential for a precise definition of EV specificity.

#### Clinical relevance

The topic of EV specificity becomes more interesting as EVs potentials are increasingly recognized in different clinical applications, particularly in the areas of targeted therapy, diagnostics, and regenerative medicine. In drug delivery, EVs can be engineered to carry therapeutic molecules directly to diseased tissue, such as tumours by exploiting their natural or modified targeting properties, thereby reducing off-target effects and improving treatment efficacy [43]. Similarly, EVs serve as valuable diagnostic tools, as their cargo reflects the physiological state of their cells of origin/the microenvironment; this enables non-invasive disease monitoring through liquid biopsies [44]. In regenerative medicine, EVs derived from specific stem cell types can selectively home to injured tissues and promote repair, offering a cell-free alternative to traditional stem cell therapy [45]. Additionally, their ability to cross biological barriers, such as the blood–brain barrier, opens new avenues for treating neurological disorders [46]. Thus, EV specificity is a critical feature that enhances their translational potential in precision medicine.

#### Technical limitations

Although EVs hold great potential for targeted therapy, several studies have observed that their in vivo target specificity is often limited [47, 48]**.** While EVs can demonstrate selective uptake in vitro, translating this specificity to complex biological systems remains a challenge. This reduced specificity is largely due to the heterogeneous nature of EV populations, which contain a mix of vesicles with diverse surface markers and cargo profiles. Additionally, systemically administered EVs are frequently sequestered by the mononuclear phagocyte system**,** particularly in the liver, spleen, and lungs, leading to off-target accumulation in vivo [49]. The lack of strong and consistent receptor-ligand interactions further hampers targeted delivery. Although surface engineering strategies such as decorating EVs with targeting peptides or antibodies have been explored, maintaining the functional integrity and targeting efficiency of modified EVs in circulation remains a significant hurdle [50]. Therefore, enhancing the target specificity of therapeutic EVs continues to be a critical area of ongoing research.

Hence, deeper understanding of the natural targeting specificity of EVs could significantly enhance the development of EV-based therapeutics. Naturally secreted EVs possess intrinsic molecular features such as specific surface proteins, lipids, and glycans, that govern their selective interaction with recipient cells [51–53]**.** By uncovering the mechanisms that underlie this innate targeting behaviour, researchers can design more precise and effective therapeutic EVs that mimic or enhance these natural properties. For instance, identifying cell type-specific ligands or tropism patterns could inform the engineering of EVs with improved homing ability to diseased tissues, thereby reducing off-target effects and increasing therapeutic efficacy. Furthermore, leveraging knowledge about how EVs interact with the immune system or microenvironment could help in customizing EV formulations for particular clinical conditions [54]. Thus, insights into natural EV specificity provide a valuable blueprint for rationally designing next-generation EV-based therapeutics**.** With this in mind, it is essential to examine whether EV targeting specificity represents a true biological phenomenon or merely a perceived concept, drawing on evidence from the current literature.

### Do EVs target cells at random?

EV uptake and the resulting functional responses have been demonstrated across a wide range of recipient cell types in the literature. Remarkably, this uptake and functionality is not limited to cells of the same origin, but it spans across species and even biological kingdoms (Table 1). This evidence suggests that EV interaction with recipient cells can occur through broadly conserved, non-specific mechanisms shared among diverse cell types.

**Table 1:** Patterns of EV–cell interactions supporting the concept of non-targeted interaction dynamics

Donor cell	Recipient cell	Functionality	Mechanism of action	Interaction pattern	Reference
***EVs mediate interactions among broad range of cell types in the same species***					
Adipose-derived mesenchymal stem cell derived EVs	Human keratinocytes and human fibroblasts	Promote proliferation, migration and protein kinase B activation in both cell types	Not known	EVs from one cell type affect multiple types of recipient cells	[55]
Human oral squamous cell carcinoma, pancreatic adenocarcinoma, and melanoma derived EVs	Normal oral gingival fibroblasts	Alter Cancer-Associated Fibroblasts (CAF)-related mRNA expression	Caused by dysregulation of Ca^2^⁺-activated ion channels due to EV uptake	EVs from different cell types affect the same cell type	[56]
Cancer associated EVs (breast, lung, kidney and glioma etc.)	Macrophages	Transform macrophage from an M1 to an M2 phenotype (indicate macrophage polarization) and support enhancing immunosuppression, angiogenesis, and metastasis	Macrophage polarization is largely driven by EV RNA cargo (including miRNAs, circRNAs, and lncRNAs) and EV protein cargo (Colony Stimulating Factor 1, Monocyte Chemoattractant Protein 1, Motif Chemokine Ligand 2 etc.) with PTEN/PI3K/AKT signaling axis frequently implicated as a key regulatory mechanism	EVs from different cell types affect the same cell type	[52]
***EVs from one species can interact with cells of another species***					
Mouse mast cells (MC/9) derived EVs	Human mast cells (HMC-1)	Influence mast cells cellular activity and differentiation	Transfer of mouse EV-derived mRNAs, which were translated into detectable mouse proteins within the human recipient cells	Functional mRNA transfer can drive cross-species translational changes when the recipient cells possess the necessary translational machinery	[57]
Bovine and human milk derived EVs	Human intestinal cells (Caco-2)	Enhance epithelial tight junction integrity	Both bovine and human milk EVs were efficiently taken up by Caco-2 cells and predominantly co-localized with lysosomes and the effect was attributed to the presence of evolutionarily conserved miRNAs	EV uptake between cells from different species is similar and effect was attributed to miRNA cargo	[58]
Bovine, human, and caprine milk-derived EVs	Human intestinal cells (Caco-2)	Significant reduction of rotaviral infection of the intestinal cells	Mechanism not very clear: antiviral activity was likely mediated by EV interactions with cellular surface receptors, either during the pre-attachment or post-attachment stages of rotavirus entry	EVs from different species bind to same recipient cells in a similar manner	[59]
***EVs can even cross kingdom barriers in communication***					
Gram negative bacterial-derived EVs	Human macrophages and epithelial cells	Activate Toll-like receptors on macrophages and epithelial cells, leading to the release of inflammatory cytokines	Gram-negative bacteria, the outer membrane contains phospholipids and glycolipids such as lipopolysaccharides (LPS), which are incorporated into bacterial EVs and bind to toll like receptors	Interkingdom communication between bacterial EVs and host cells is driven by the molecular cargo carried by the EVs and the receptor repertoire of the host cells	[60, 61]
Plants cells derived EVs	Fungal cells	Degrade fungal cell walls and protect against infection	Plant-derived EVs carry antifungal enzymes capable of degrading fungal cell walls	Conservation of certain EV-associated protein functions across kingdoms	[62, 63]
Human cell-derived EVs	Fungal pathogen *Aspergillus fumigatus*	Accumulate in large numbers on the hyphal cell membrane	Not known	Human cell derived EVs binding to fungal cells	[64]

EVs mediate communication among a wide range of cell types within the same species. This interaction can occur in two major ways: EVs from single cell type can act on multiple recipient cell types to induce similar functional effects [55], or EVs originating from different cell types can elicit same effect on a common recipient cell type [56]. In both scenarios, the observed outcomes are largely driven by the molecular cargo of EVs, which can either interact with receptors on the recipient cell surface or be internalized to release their contents and trigger downstream signalling pathways. EV-mediated communication can also extend across species. EVs derived from cells from one species are capable of interacting with, binding to, or being internalized by either similar or entirely different types of recipient cells in another species [57, 59]. The functional outcomes in such cross-species interactions similarly depend on the transfer of EV cargo. For instance, the delivery of functional mRNA from cells from one species can drive translational changes in recipient cells in other species, provided that the necessary translational machinery is present in the recipient cell [57, 65]. Interestingly, EV uptake or binding mechanisms often remain conserved across species, enabling EVs from one species to interact with recipient cells of another species [6, 66]. In addition to that, bacterial, parasitic and plants cells are also known to release EVs that can penetrate the rigid cell walls of other plant cells, microbial pathogens as well as mammalian cells [62]. This cross-kingdom communication contributes to plant defence against microbial pathogens and symbiotic interactions among plants and microbes. Such cross-kingdom uptake where EVs from human cells can be internalized by animal, plant, bacterial, fungal, or parasitic cells (and vice versa) highlights the remarkable versatility of EVs as mediators of interspecies communication and carriers of molecular information [63, 67, 68]. As discussed above, cross-species and cross-kingdom interactions may result from the transfer of functional molecular cargo of EVs to diverse recipient cells. Many of these molecular components are conserved across species and even across kingdoms enabling EVs to elicit similar functional effects even in phylogenetically distant cells. Despite their broad activity, EVs can also display target specificity. The next section highlights cases of such selective interactions and the factors that govern them.

### Do all cells respond to EV messages equally?

Interestingly, ample evidence in the literature suggests that not all cells respond equally to EV signals. This differential response of recipient cells to EVs can be detected at uptake level or functional response level. EV specificity can range from a complete lack of interaction with certain cells to varying degrees of interaction efficiency. In Table 2, we summarized studies demonstrating diverse patterns of EV-recipient cell interactions, highlighting that specificity may appear at the uptake level (binding, internalization, or cargo release), at the functional level, or at both. To gain deeper insight into the mechanisms of EV targeting, we used Table 2 to examine distinct patterns of EV-target cell specificity.

**Table 2:** Recipient cells exhibit differential responses to extracellular vesicles (EVs), evident at the levels of EV binding, internalization, cargo release and functional outcomes

EV and recipient cell type Evidence of EV specificity	Method of detection of EV interaction with recipient cell	Potential mechanisms of EV target specificity	Tropism is defined at the level of uptake/functionality?			Reference
**EV type:** EVs originated from mesenchymal stem cells/fibroblasts, epithelial cells, immune cells and amnionic fluid cells **Recipient cell type:** fibroblasts **Observation of specificity:** EVs from different cell sources produce specific transcriptional responses in recipient fibroblasts. These cellular responses corresponded with the activities and phenotypes of the EV source cells	Functionality of each EV type was analyzed using recipient cell transcriptomic changes	Specific combination of EV and cell type EV internal cargo and surface proteome EV dose also decides specific functionality	**Uptake**			[16]
			Binding	No		
			Internalization	No		
			Cargo release	No		
			**Functionality**			
			Change of function **Specificity type: Only functional specificity was shown**	Yes		
**EV type:** K7M2 cell-derived EVs and NIH/3T3 cell-derived EVs produced under various incubation times (12, 24, 48, and 72 h) and temperatures (37 °C and 40 °C) **Recipient cell type:** K7M2 and NIH/3T3 **Observation of specificity:** Nutrient stress enduring cell secreting K7M2-72 h EVs showed pro-proliferative functional impact while heat stress enduring cells secreting K7M2-40C EVs showed the anti-proliferative functional impact this functional impact was observed in both cancer and non-cancer cells, it was more prominent in cancer cells K7M2	Functionality of the EVs were analyzed by cell proliferation assay as the end point of EV activity	Specific combination of EV and cell type Effect of cellular microenvironment and EV protein cargo likely affects the EV functionality	**Uptake**			[69]
			Binding	No		
			Internalization	No		
			Cargo release	No		
			**Functionality**			
			Change of function **Specificity type: Only functional specificity was shown**	Yes		
**EV type:** HEK293T EVs **Recipient cell/tissue type:** HEK 293 T cells, epithelial (C3A liver cells), endothelial (human umbilical vein endothelial cells), and neural (SH-SY5Y glioblastoma cells, human neural stem cells (hNSCs) and mature human neurons **Observation of specificity:** All the cell types internalized HEK293T EVs but increased uptake was detected by HEK293T cells. Neural stem cells internalized more EVs compared to mature human neurons	GFP labelled HEK293T EV uptake inside different recipient cells were determined by flow cytometric methods	Self-cell selectivity Differentiation status of recipient cells (Mature cells take up less EVs)	**Uptake**			[70]
			Binding	No		
			Internalization	Yes		
			Cargo release	No		
			**Functionality**			
			Change of function **Specificity type: Only uptake specificity was shown**		No	
**EV type:** glioblastoma-cell (U251) derived EVs and astrocyte derived EVs **Recipient cell/tissue type:** U251, breast cancer, fibrosarcoma**,** astrocytes **Observation of specificity:** U251-derived EV uptake was highest in the U251 cells, followed by breast cancer cells, fibrosarcoma and astrocytes	PKH67 labelled EV internalization in to recipient cells was detected by confocal microscope and flow cytometry methods	Self-cell selectivity	**Uptake**			[71]
			Binding		No	
			Internalization		Yes	
			Cargo release		No	
			**Functionality**			
			Change of function **Specificity type: Only uptake specificity was shown**		No	
**EV type:** Jeko-1 EVs, Mino-EVs, MCL EVs (Mantle cell lymphoma) **Recipient cell/tissue type:** Jeko-1, Jurkat, HS-5 cells, Mino-cells, T-lymphocytes, B-Lymphocytes, NK cells, monocytes, **Observation of specificity:** Jeko-1 EVs preferentially internalized Jeko-1 cells, with no apparent internalization into Jurkat and HS-5 cells. Mino exosomes were uptaken rapidly and preferentially by Mino cells MCL EVs internalized by B-lymphocytes more efficiently as opposed to healthy T-lymphocytes and NK cells that barely internalized MCL EVs	PKH-26/PKH-67 labelled EV uptake was measured by confocal microscopy and flow cytometry	Self-cell selectivity Specific combination of EV and recipient cell	**Uptake**			[72]
			Binding		No	
			Internalization		Yes	
			Cargo release		No	
			**Functionality**			
			Change of function **Specificity type: Only uptake specificity was shown**		No	
**EV type:** two melanoma cell lines (A375, 1205Lu) and one ovarian cancer cell line (OvC16) derived EVs **Recipient cell/tissue type:** A375 cells **Observation of specificity:** A375 cells cell derived EVs were taken up by A375 cells with similar efficiency as EV produced in other cancer cell lines Macrophages and mature dendritic cells take up EV more efficiently than monocytes or immature dendritic cells	EVs were fluorescently labelled by two different dyes, carboxyfluoresceine diacetate succinimidyl-ester (CFSE) uptake was monitored by flow cytometry and immunofluorescence microscopy in a competitive uptake environment	Maturation status of cell (Mature cells take up more EV)	**Uptake**			[73]
			Binding		No	
			Internalization		Yes	
			Cargo release		No	
			**Functionality**			
			Change of function		No	
			**Specificity type: Only uptake specificity was shown**			
**EV type:** Human mesenchymal stem cell (MSc) derived EVs and fibroblast derived EVs **Recipient cell/tissue type:** Mice kidney **Observation of specificity:** MSc EVs could protect mice kidney against glycerol induced acute kidney injury compared to human fibroblast EVs MSc EVs were preferentially accumulated in kidney and liver compared to lung tissue	Protection from acute kidney injury was determined by blood urea nitrogen and creatinine level and histology in mice PKH26 labelled EV accumulation in mice organs were detected by confocal microscopy	Specific combination of EV and cell type (cross species)	**Uptake**			[74]
			Binding		No	
			Internalization		Yes	
			Cargo release		No	
			**Functionality**			
			Change of function **Specificity type: Specificity was shown at both uptake and functional level and correlated but not experimentally validated**		Yes	
**EV type:** Chronic lymphocytic leukemia (CLL) derived EVs, MEC-1 cell derived EVs **Recipient cell/tissue type:** Bone marrow (BM)-derived stromal cell line HS-5 and the endothelial cell line HMEC-1 **Observation of specificity:** CLL- EVs were up taken specifically by BM-stromal, endothelial and myeloma cells, but not by CLL B cells MEC-1 exosomes were taken up by, endothelial, stromal, and multiple myeloma cells, but not CLL cells In vivo administration of CLL EVs caused EV uptake in liver, spleen and peripheral blood of mice. CLL EVs but not healthy donor B cell EVs were able to induce specific cell signaling pathways leading to transformation of stromal and endothelial cells to have CAF (cancer associated fibroblast) phenotype and other cancer cell properties	PKH67-labeled CLL-EVs internalization was detected with confocal microscopy Functional specificity was detected by signaling pathway activation	Specific combination of EV and cell type Specific receptors on recipient cells	**Uptake**			[75]
			Binding		Yes	
			Internalization		Yes	
			Cargo release		No	
			**Functionality**			
			Change of function **Specificity type: Specificity was shown at both uptake and functional level and correlated but not experimentally validated**		Yes	
**EV type:** Ectosomes from neutrophils and liposomes **Recipient cell/tissue type:** HUVEC, red cells, THP-1 cells **Observation of specificity:** Ectosomes from neutrophils bound to both THP-1 (monocytic cells) and HUVEC cells (endothelial cells) but not to red blood cells in vitro. Liposomes bound to all recipient cell types	Ectosomes were labeled with PKH67 dye and analyzed with Fluorescence activated cell scanning (FACScan) and confocal microscopy	Specific combination of EV and cell type Adhesion protein in ectosome surface (L-selectin)	**Uptake**			[76]
			Binding		Yes	
			Internalization		Yes	
			Cargo release		No	
			**Functionality**			
			Change of function **Specificity type: Only uptake specificity was shown**		No	
**EV type:** EVs from neuroblastoma cells and cortical neuron derived EVs **Recipient cell/tissue type:** neurons, astrocytes and oligodendrocytes **Observation of specificity:** Neuroblastoma EVs bound nonspecifically to both neurons and glial cells, but endocytosed preferentially by glial cells whereas stimulated cortical neuron derived EVs were bound and endocytosed only by neuronal cells but not by glial cells	GFP-CD63 labelled EV binding and internalization were detected under fluorescent microscopy	Specific combination of EV and cell type	**Uptake**			[77]
			Binding		Yes	
			Internalization		Yes	
			Cargo release		No	
			**Functionality**			
			Change of function **Specificity type: Only uptake specificity was shown**		No	
**EV type:** Trophoblast cell derived EVs and HEK293T cell derived EVs **Recipient cell/tissue type:** Endometrial epithelial cells (RL95-2 cells) **Observation of specificity:** Trophoblast derived EVs specifically reprogram endometrial epithelial cells (RL95-2 cells) transcriptome towards embryo implantation and HEK293T cell EVs did not cause any transcriptomic changes	Effect of EV on recipient cell transcriptome was detected	Specific combination of EV and cell type	**Uptake**			[78]
			Binding		No	
			Internalization		No	
			Cargo release		No	
			**Functionality**			
			Change of function **Specificity type: Only functional specificity was shown**		Yes	
EV type: Bovine follicular fluid derived EVs, human trophoblast derived EVs and porcine follicular fluid derived EVs **Recipient cell type:** Sperm **Observation of specificity:** Bovine follicular fluid derived EVs increased bovine sperm viability, capacitation and acrosome reaction. However, human trophoblast derived EVs and porcine follicular fluid derived EVs were unable to induce any changes. Trypsin treatment to modify EV surface caused EV functional loss	Change of recipient cell function was detected using functional assays	Specific combination of EV and cell type Surface protein cargo has a role in specific EV interaction with selected recipient cells	**Uptake**			[79]
			Binding		Yes	
			Internalization		No	
			Cargo release		No	
			**Functionality**			
			Change of function **Specificity type: Only functional specificity was shown**		Yes	
**EV type:** Organotrophic (human lung, liver and brain) human breast and pancreatic cancer cell derived EVs **Recipient cell/tissue type:** lung, liver and brain cells in mice **Observation of specificity:** exosomes from mouse and human lung, liver- and brain-tropic tumour cells fuse preferentially with resident cells at their predicted destination	EV binding was assessed by confocal microscopy Bio distribution analysis was performed using near infrared or red fluorescently labelled EV EV functional specificity was analysed by activation of specific signalling pathways	Specific combination of EV and cell type EV organotropism was guided by EV surface integrin signature	**Uptake**			[80]
			Binding		Yes	
			Internalization		No	
			Cargo release		No	
			**Functionality**			
			Change of function **Specificity type: Specificity was shown at both uptake and functional level and correlated with experimentally validated data**		Yes	
**EV type:** Exosomes and microvesicles from Ovalbumin pulsed dendritic cells **Recipient cell type:** antigen-specific CD8^+^ T-cells **Observation of specificity:** Exosomes were more efficient in eliciting antigen-specific IgG production in CD8^+^ T-cells compared to microvesicles derived from ovalbumin (OVA)-pulsed dendritic cells, suggesting exosomes might have tropism towards dendritic cells	Detection of antigen-specific IgG production	Effect of different EV subpopulations	**Uptake**			[81]
			Binding		No	
			Internalization		No	
			Cargo release		No	
			**Functionality**			
			Change of function **Specificity type: Only functional specificity was shown**		Yes	
**EV type:** human bone marrow mesenchymal stromal cells (MSCs) exosome s and microvesicles **Recipient cell type:** EV population enriched with exosomes were able to induce renal regeneration in acute kidney injury both in vivo and in vitro whereas EV population enriched with microvesicles were unable to exert the same effect	Renal function and morphology were assessed	Effect of different EV subpopulations Molecular cargo content of exosom es and macrovesicles are different	**Uptake**			[82]
			Binding		No	
			Internalization		No	
			Cargo release		No	
			**Functionality**			
			Change of function **Specificity type: Only functional specificity was shown**		Yes	

### Patterns of EV specificity

*EVs are naturally attracted to their cell of origin;* Many studies have demonstrated that EVs often exhibit a preferential affinity for their cells of origin, a phenomenon referred to as "EV homing." For example, tumour-derived EVs show a strong preference for interacting with other tumour cells of the same type rather than benign cells of the same origin both in vitro and in vivo [83]. This selectivity is linked to surface-mediated signalling—such as engagement of EV surface associated heparan sulfate proteoglycans (HSPGs) [84] or the potential transfer of functional RNA or protein cargo through dynamin-dependent endocytosis [85]. Interestingly, malignant cancer cells preferentially interact with other malignant cells of the same origin, compared to primary tumour or benign cells of the same origin. This selectivity has been associated with the cell cycle, as malignant cells in highly metabolically active phases internalize substantially more EVs. These uptake patterns correlated closely with increased proliferative capacity in malignant cells, although they do not align with changes in their migratory behaviour [86]. Nevertheless, phenomenon of tumour EV homing appears to be quite broad. Human tumour cell-derived EVs have been shown to target neoplastic tissues in mice, suggesting that EV homing can occur independently of tumour type or even species of origin [87]. However, the concept is not without exceptions [88, 89]. For instance, EVs derived from breast cancer cells exhibited less uptake by their parental cells compared to HEK293T cells when EV uptake was measured using fluorescent based assay [88]. This anomaly was later attributed to the high efficiency of fluorescent labelling in HEK293T-derived EVs, which can result in artificially elevated fluorescence signals. This highlights the importance of caution when interpreting EV uptake data. In addition to cancer cells, immune cell-derived EVs also show target specificity towards cells of their origin both in vivo and in vitro. Dendritic cells (DC) preferentially interact with other DCs, supporting rapid T-cell activation and a robust immune response [63]. DC EVs have been shown to accumulate in the spleen following systemic administration, indicating organ-specific homing of immune cells in vivo [67]. However, whether this preferential uptake leads to consistent functional consequences is not well established in all cases.

In summary, EVs often exhibit a preference for their cells of origin, which is observed both at the level of uptake and functional impact in many cases. It is also critical to distinguish between in vitro and in vivo observations. In vitro systems often display clear cell-type-specific uptake due to their simplicity. In contrast, in vivo EV homing is influenced by a multitude of factors including the route of administration, recipient animal type, EV dose, EV subtype, and the timing of distribution assessment [90]. Therefore, conclusions about EV specificity in vivo must be made carefully. Nonetheless, EV uptake alone does not always guarantee functional relevance and uptake itself is not always correlated well with the functional response. The EV tropism towards parent cell is regulated by a combination of factors, including the EV protein and RNA cargo, receptor-ligand interactions, recipient cell surface characteristics, cell cycle stage, and overall cellular context. However, several important questions remain unanswered: Why does EV homing occur? Why are EVs preferentially taken up by the cells that released them, and how these phenomena influence the function of those cells? What is the biological significance of this behaviour for the originating cell? Could this be a strategy used by cancerous or similar cell types to communicate and coordinate with each other, much like bacteria that use vesicles for intercellular communication and transfer of antibiotic resistance? Is EV-mediated communication a means of information exchange that enhances collective survival? Uncovering the mechanisms that drive EV attraction to their parent cells could provide valuable insights for improving both therapeutic and diagnostic applications.

*Immune cells show selectivity in interacting with EVs;* Several immunology studies have highlighted the existence of specific interactions between EV and immune cell membranes that contribute to EV uptake specificity. For example, human milk-derived EVs containing the MUC1 ligand have been shown to interact with DC-SIGN, a pathogen-recognition receptor on monocyte-derived DCs. This interaction inhibits HIV transmission, an effect not observed with plasma-derived EVs [91]. EVs derived from rat pancreatic adenocarcinoma cells were internalized by various leukocyte types in vivo, but with varying levels of efficiency. DCs and macrophages exhibited the highest uptake, while granulocytes showed the lowest. Notably, blocking the tetraspanins CD9 and CD81 significantly reduced tumour EV binding to immune cells [92]. In this context, EV binding triggered distinct functional responses depending on the recipient immune cell type. However, the extent to which differences in EV uptake corresponded to functional differences was not demonstrated. EVs derived from multiple myeloma cell lines (MM.1S, H929, and U266) were internalized by monocytes, with H929 EVs showing the highest uptake. This was accompanied by a marked increase in monocyte secretion of MMP-9 and IL-6, with H929 EVs inducing the strongest response in line with their higher uptake levels. This effect was linked to the enrichment of spliceosome-associated proteins in H929 EVs [93]. These findings illustrate that EV binding to recipient cell membranes can involve specific ligand-receptor interactions, which not only influence the route of uptake but also contribute significantly to EV targeting and functional specificity.

*Specific EV and recipient cell pairs exists in many other cases where EV communication is critical;* In a recent study, we demonstrated that EVs derived from trophoblast cells specifically induced gene expression changes in receptive endometrial epithelial cells, but not in non-receptive endometrial epithelial cells or non-endometrial cells. Furthermore, EVs from unrelated cell types, such as HEK293T cells, failed to induce any transcriptomic changes in receptive endometrial cells, underscoring the specificity of EV-recipient cell interactions [9]. These variations were attributed to differences in EV RNA, protein cargo, and surface proteome composition [94, 95]. This unique communication between trophoblast and receptive endometrial cells is critical for the process of embryo implantation. Supporting this, another study showed that when 12 different EV types were applied to fibroblasts, each EV-recipient cell pair produced a distinct transcriptomic response [16]. Certain EV types, such as those derived from mesenchymal stem cells, have also been shown to exhibit strong tropism toward damaged or neoplastic tissues and repair them, highlighting their targeted behaviour in complex multicellular environments [96, 97].

In the literature, we identified four main scenarios in which EVs can be referred to as “specific.” In the first scenario, *certain EV types are preferentially uptaken by specific cell types*. Although most cells with active endocytic pathways can internalize EVs to some degree, the efficiency of uptake may vary widely. The limitation of relying solely on uptake measurements is that internalization does not necessarily indicate functional cargo delivery. EV cargo can be retained within endosomes, recycled back to plasma membrane, or degraded in lysosome without activating any signalling events [11, 98, 99]. In some cases, EV internalization is not required at all for functional response (e.g. Activation of T cell receptors) [100]. A recent study showed that while EVs can bind to both self and non-self-cells, activation of downstream signaling is observed only in non-self-cells [101]. Additionally EV uptake is commonly measured using fluorescence labelling of EV membrane or cargo followed by detection with microscopy or imaging flow cytometry [5, 89, 102]. These methods provide information on the extent to which EVs are internalized or bound to recipient cells, but can also produce misleading results due to issues such as fluorescent dye leakage across the plasma membrane or fluorescent dye aggregates being mistaken for EVs. Above evidence also suggest EV uptake selectivity doesn’t necessarily mean functional selectivity. Therefore, determining EV target specificity solely on the basis of uptake/EV internalization, as is commonly done in the literature, can be misleading. In the second scenario, *uptake specificity is not demonstrated, yet functional specificity is evident.* Functional assays assess the biological effects triggered by EVs in recipient cells, such as changes in gene expression, cytokine release, or activation of specific signaling pathways [9, 16, 78, 103]. In these cases, EVs elicit distinct biological effects only in certain recipient cell types, and EV uptake can be random or selective. In the third scenario, *EV uptake shows specificity, but this does not translate into corresponding functional specificity* [86]. In the fourth scenario*, specificity is observed at both the uptake and functional levels,* indicating that EV-cell interactions are shaped by a combination of physical targeting mechanisms and cell-specific biological responses [80].

While EV specificity clearly exists, the exact mechanisms remain unclear. Referring to EVs as “specific” in all scenarios is not always justified, particularly when specificity is inferred solely from uptake. To truly understand how cells drive EV specificity, both uptake and functional dynamics should be considered; however, to make specificity biologically meaningful, functional outcomes should serve as the primary endpoint. Importantly, EV-mediated intercellular communication appears to be finely tuned according to both the origin of the EVs and the identity of the recipient cells.

### What factors tune the EV interaction with the selective recipient cells?

Building on this overview, we highlight key factors that influence and modulate EV interactions with specific recipient cell types. Importantly, most studies define EV specificity only up to the point of internalization, with relatively few extending this to demonstrate functional specificity. It is also noteworthy that the majority of studies were not specifically designed to investigate selective EV interactions with recipient cells; instead, such observations were often reported incidentally or as secondary findings.

#### The role of recipient cell machinery in mediating selective EV uptake during EV-cell interactions

The EV uptake process might follow sequential steps starting from EV direct interaction with the recipient cell membrane and fusion to internalization. EV internalization is considered a nutritive function, or molecule recycling mechanism, inside the recipient cells. Cells appear to internalize EVs through various endocytic pathways, including clathrin dependent endocytosis, clathrin-independent pathways such as caveolin-mediated uptake, micropinocytosis, phagocytosis, or lipid raft–mediated internalization [5]. Given the fact that EVs are internalized by many cell types and the complex heterogeneous nature of the EVs, it is possible that a population of EVs may gain entrance to the recipient cells via more than one route.

EV uptake begins with fusion to the recipient cell membrane. Prada et al. (2016) used optical tweezers to place single microglia-derived EVs onto microglia or astrocytes in vitro. They found that EVs displayed more directional movement on microglial membranes, while movement on astrocytes was random. This suggests that EV tropism can begin at the point of membrane binding, likely mediated by surface receptors or extracellular ligands [104]. On the other hand, because EVs are nanosized and have a lipid bilayer, they can be easily taken up by many cell types. Clathrin and caveolin mediated endocytosis are two main energy dependent routes of EV internalization in to cells. In some recipient cells, specific endocytic pathways are more active than others. For example, when EVs from HCT116, A549, and COLO205 cells were introduced to these same three cell types as recipient cells, EV uptake was highest in HCT116 cells, followed by A549 and COLO205, regardless of the donor EV type. The higher uptake observed in HCT116 cells was associated with elevated caveolin-1 mRNA and protein levels compared to A549 cells, while Caveolin-1 was absent in COLO205 cells. Since Caveolin-1 and clathrin heavy chain are key mediators of caveolae- and clathrin-dependent endocytosis, the greater EV uptake in HCT116 cells was attributed to a more active endocytic machinery [105]. Additionally, reports suggest that phagocytic cells internalize more EVs compared to non-phagocytic cells. Feng and colleagues demonstrated that EVs derived from K562 or MT4 cells were internalized more efficiently by professional phagocytic cells — including macrophages, U937 monocyte-derived macrophages, and THP-1 myelomonocytic cells — than by non-phagocytic cells such as mouse fibroblasts, human lung fibroblasts, Jurkat T cells, 293 T cells, and COS-7 kidney cells. In phagocytic cells, EV uptake was dependent on the actin cytoskeleton phosphatidylinositol 3-kinase, dynamin 2, and the phagocytic receptor TIM-4, while pathways such as caveolae-mediated uptake, macropinocytosis, and clathrin-mediated endocytosis were not involved. In contrast, in non-phagocytic cells, EVs merely bound to the cell surface without internalization. These findings suggest that certain cells are equipped with specialized machinery for EV internalization [106]. Another interesting observation highlighting the role of endocytic machinery in EV uptake was shown in a study where ectosomes from neutrophils bound to both THP-1 monocytic cells and HUVEC endothelial cells, but not to red blood cells in vitro*.* Since mature red blood cells lack endocytic capability, they were unable to internalize EVs [76]. Once internalized via endocytic routes, EVs follow typical endosomal pathway, where early endosomes undergo acidification to become late endosomes or MVBs and fuse with lysosomes for degradation [107].

Although EVs that fuse directly with the plasma membrane can deliver their cargo immediately into the cytosol, cargo release from EVs that enter cells via endocytosis appears to be a rate-limiting step for their functionality. Factors such as cholesterol accumulation and pH changes within endosomes can significantly influence cargo release efficiency. For example, in HEK293T cells, cholesterol-dependent changes in endosomal pH have been shown to regulate EV cargo release [98, 108]. Following uptake, EVs first localize to early endosomes, which typically mature toward lysosomal degradation, although EVs can also be recycled back to the plasma membrane. The phenotypic changes observed in recipient cells indicate that EV cargo can evade lysosomal breakdown. Current evidence suggests that endosomal membrane fusion is the main mechanism by which EVs release their cargo into the cytosol. Endosomal fate is influenced by late-endosomal cholesterol content, Rab GTPases, and SNARE protein expression, which regulate whether vesicles fuse with lysosomes, recycle to the plasma membrane, or become permeabilized to release cargo [12, 107, 109]. Rab proteins such as Rab7, Rab11, and Rab27 are key determinants of this trafficking, directing late endosomes toward lysosomal fusion, recycling routes, or secretion [110]. However, how these pathways contribute to cargo release in specific EV-cell interactions remain poorly defined. In most of the cases described above, it remains unclear whether increased EV uptake consistently leads to enhanced functional outcomes.

#### Role of the EV surface proteome in selective EV interaction with recipient cells

The mechanism by which EVs are taken up by recipient cells is closely influenced by the membrane composition of both the EVs and the target cells [111]. This process is largely governed by the type and abundance of surface proteins, lipids, and glycoproteins present on the vesicle membrane [112]. Key surface molecules on EVs—such as tetraspanins, integrins, extracellular matrix proteins, lectins, proteoglycans, and members of the immunoglobulin superfamily—play critical roles in mediating EV-target cell interactions and can contribute to the observed tropism of EVs toward specific cell types. These surface components are not only essential for cellular recognition, docking and internalization of EVs, but can also influence downstream functional outcomes following EV uptake [113].

Tetraspanins on the EV surface not only facilitate EV binding to recipient cell membranes but also interact with and remodel extracellular matrix proteins [89, 101]. Tetraspanins, such as Tspan8, in EVs are crucial for their exit from circulation and for guiding their targeted accumulation in specific organs in vivo [114]. In the context of pancreatic cancer, EVs displaying CD9 on their surface can bind to p38 receptors on tumour stromal cells, activating the mitogen-activated protein kinase (MAPK) signaling pathway and promoting cell migration [115]. These findings highlight that the role of EV surface tetraspanins in uptake and functionality is highly dependent on the specific pairing between EVs and recipient cells [116]. However, not all interactions follow this pattern. EV surface CD9 and CD63 had shown no effect on EV internalization or cargo release within the same cancer cell type [89, 116].

In the tumour microenvironment, adhesion molecules on tumour-derived EVs help determine which cell types they interact with. For example, intercellular adhesion molecule-1 (ICAM-1) on EVs from human cancer cells can bind to leukocytes and inhibit their adhesion to endothelial cells, thereby modulating the immune response within the tumour microenvironment [117]. Additionally, cell surface integrins such as α6β1/α6β4 and α2β1, along with the ganglioside GM1 have shown to mediate tumour cell binding to endothelial cells in laminin dependent manner and influence their morphogenesis [101]. Specific integrin patterns on tumour-derived EVs have been associated with organ-specific metastasis. For instance, α6β1 and α6β4 integrins promote EV targeting to the lungs, whereas αvβ5 directs EVs to the liver to form the pre-metastatic niche [80]. During inflammation, EV surface molecules are important for directing specific cellular interactions. For instance, activated Integrin β1 on hepatocyte-derived EVs promotes monocyte adhesion to liver sinusoidal cells which is an important step in mediating liver inflammation [118].

Additionally, EV surface glycans also play a role in EV specific interaction with target cells [119]. For example, when adipose-derived stem cell EVs were glycoengineered to display four distinct surface glycans (sialic acid, galactose, GlcNAc, or mannose), their uptake by different cell types and their biodistribution in vivo varied according to the specific glycan pattern [120]. Hence, EV surface proteome regulate cell–cell interactions through ligand-receptor pairing between EVs and recipient cells, thereby influencing EV binding, internalization, cargo delivery, and the activation of downstream signaling pathways.

#### The role of the EV protein corona in selective EV interaction with recipient cells

EV interactions with recipient cells are also influenced by the EV corona, a layer of biomolecules, primarily proteins, lipids, and other solutes, that adsorb onto the EV surface upon exposure to biological fluids [121]. This dynamic biomolecular layer can significantly modify the native surface characteristics of EVs, thereby affecting their recognition, cellular uptake, and functional outcomes [122]. For instance, corona of EVs derived from therapy-grade human placental-expanded stromal cells induced differential signalling in immune cells, suppressed T cell proliferation in vitro, and enhanced skin regeneration and angiogenesis in vivo [123]. Cancer cells exploit a similar strategy. Tumour cell-derived EVs (HCT116), when coated with a protein corona, exhibited enhanced uptake by different immune cells with monocyte-derived dendritic cells showing the highest internalization [124]. Even the immune system leverages this mechanism. Human immune cell-derived EVs (from THP-1 monocytes) can acquire a coating of plasma proteins in the blood, including ApoA1, ApoB, ApoC3, ApoE, and complement factor 3, which enhances their uptake and the cytokine secretion from human monocyte-derived dendritic cells, amplifying the immune response [125]. These coronas are not passive coatings—they actively mediate ligand-receptor interactions, triggering signaling pathways that can shape cell behaviour. For examples, tumour EV corona made of fibronectin bind to heparan sulfate-binding ligand in target cells to activate signalling pathways that facilitate cancer progression and invasion [126]. Another example is human pluripotent stem cells (hPSC) derived EV-associated corona protein MFGE8 which activate signalling pathways that enhances EV internalization and hPSC growth [127]. Moreover, protein corona derived from host proteins may facilitate tissue-specific targeting of EVs in vivo [128].

These findings emphasize that the EV corona plays a critical role in determining EV surface properties, stability, biodistribution, and targeting efficiency. Importantly, the corona’s composition is not static, it varies according to the biological fluid environment (e.g., plasma, cerebrospinal fluid, or conditioned media), the surface characteristics of the EV, and the specific EV subtype [124, 129]. Thus, both the intrinsic EV membrane and the acquired corona must be considered when evaluating EV-cell interactions, therapeutic targeting, and functional outcomes in vitro and in vivo.

#### Recipient cell differentiation status in EV selective interaction with recipient cells

The differentiation and maturation status of recipient cells significantly influences their interaction with EVs. For example, EVs released by immature DCs show functional differences based on size: large EVs tend to promote a Th2 cytokine response, while small EVs preferentially induce Th1 responses, such as IFN-γ secretion. Interestingly, these functional distinctions disappear once DCs mature, at which point both EV subtypes uniformly promote IFN-γ production [130]. Ectosomes derived from neutrophils can bind to THP-1 monocytes and HUVEC endothelial cells in vitro but not to mature red blood cells, likely because mature erythrocytes lack the endocytic machinery necessary for EV internalization [76]. This trend is also evident in the nervous system: neural stem cells have a significantly higher capacity to internalize EVs compared to mature human neurons [70]. This is believed to result from the highly proliferative nature of immature cells, which may non-specifically take up both nutrients and EVs, although this has yet to be conclusively demonstrated. These findings suggest that the cellular differentiation stage plays a critical role in modulating EV uptake and subsequent functional responses.

#### Effect of cellular microenvironment in EV selective interaction with recipient cells

The microenvironment of donor cells plays a crucial role in shaping the composition and functional properties of the EVs they release. Consequently, EVs can induce functional effects with varying intensity depending on these conditions [21, 22]. In particular, microenvironmental factors such as cytokines have been shown to modulate EV behavior. For instance, the cytokine CCL2 can bind to glycosaminoglycan side chains on cancer-derived EVs, effectively decorating their surface. This modification promotes the selective accumulation of EVs in specific cell types and organs, ultimately altering the immune landscape and contributing to increased metastatic burden [54].

Hypoxic microenvironment, in particular, has been shown to enhance EV uptake. Hypoxia-induced EVs not only exhibit higher uptake rates compared to normoxic EVs but also promote motility, invasiveness, and stemness in primary tumour-derived cells, although they exert limited effects on metastasis-derived cells [131]. Interestingly, hypoxic cardiomyocytes have been shown to enhance the uptake of EVs derived from adipose-derived regenerative cells via clathrin-mediated endocytosis compared to normoxic cardiomyocytes. This effect is driven by reactive oxygen species generated during hypoxia, which phosphorylate AP2, promoting clathrin assembly and activating clathrin-mediated endocytosis, thereby facilitating more efficient EV internalization and cargo delivery [132]. Nevertheless, cargo delivery efficiency of mesenchymal stem cell-derived small EVs was significantly reduced under hypoxic conditions in inflammaging nucleus pulposus cells. This reduction was attributed to the activation of endocytic recycling pathways, which hinder effective cargo delivery [133]. Interestingly, hypoxic EVs has been shown to enhance cell proliferation and blood‐brain barrier permeability in cancer cells but not in non-cancerous cells [134]. In this context, EV tropism was attributed to elevated VEGF-A expression on the surface of hypoxic EVs, which disrupts claudin-5 and occludin expression in the plasma membrane of cancer cells. In experimental set-ups, the physical properties of the culture environment also influence EV uptake. For example, cells cultured on soft matrices showed significantly enhanced EV internalization compared to those grown on rigid tissue culture polystyrene surfaces, but exact mechanism is not clear [88]. Furthermore, differences in EV functionality have been observed between cells grown in traditional 2D monolayers versus 3D cultures, underscoring the importance of cell dimensionality in EV biology. The functional differences was mainly attributed to EV cargo profile differences [21, 22].

These findings suggest that various microenvironmental factors—such as cellular stress, extracellular matrix (ECM) composition, and cellular architecture—can significantly alter EV composition and functionality.

#### Role of EV physicochemical characteristics in EV selective interaction with recipient cells

Several physicochemical factors influence the efficiency and speed of EV uptake by recipient cells. One key determinant is particle size. Nanoparticles, including EVs smaller than 200 nm, are preferentially internalized via clathrin- and caveolin-mediated endocytosis, highlighting the impact of size on uptake mechanisms [135]. In addition to size, surface characteristics such as charge and molecular composition also play a crucial role [124]. These features influence the strength of electrostatic interactions of EVs with the recipient cell membrane, thereby affecting uptake pathways, subcellular localization, and delivery efficiency. For instance, the introduction of lipophilic functional groups to nanocarrier membranes has been shown to shift their uptake mechanism predominantly toward lipid raft-mediated endocytosis, underscoring the importance of membrane composition [136]. However, it is still unclear whether adding functional groups to the EV membrane can affect the natural functionality of EVs. Biodistribution patterns also vary with EV size. Large EVs predominantly localize to the lungs shortly after administration, peaking within the first hour and declining between 2–12 h. In contrast, small EVs tend to accumulate in the liver, with peak detection occurring in the liver and kidney within the first hour, and subsequent distribution to the lungs and spleen between 2–12 h [90]. However, size does not always dictate function. For example, large EVs released by human DCs have been shown to be just as effective as small EVs, including exosomes in activating CD4 + T cells in vitro [130]. Due to the current limitations in isolating and characterizing distinct EV subtypes, it remains challenging to comprehensively study the subtype-specific functional impacts of EVs. Continued methodological advancements are necessary to better elucidate these relationships.

#### Role of molecular cargo in selective EV interaction with recipient cells

EVs carry a diverse array of molecular cargo, including RNA, proteins, lipids, DNA, and other metabolites reflecting the molecular profile of their parent cells. This cargo plays a key role in determining EV functionality. There is ample evidence to say EV cargo molecules participate in exerting distinct biological effects within recipient cells (Table 3). Below, we discuss how EV RNA, proteins, and lipids contribute to modulating EV interactions with recipient cells.

**Table 3:** Extracellular vesicle cargo molecules mediate specific functions in recipient cells

Source of EVs	Recipient cells	Studied cargo composition	Effect on recipient cells	Mechanism of action	Reference
Chronic lymphocytic leukaemia derived EVs	Endothelial and mesenchymal stem cells	RNA and protein cargo: miRNAs such as miR-150, miR-146a and miR-155 Protein Cargo: human leukocyte antigen (HLA)-DR, B cell specific markers (CD19 and CD20), tetraspanins (CD37, CD53, and CD82)	Activate multiple signalling pathways including but not limited to AKT, phosphorylation and (NF)-κB signaling. Induces an inflammatory phenotype in the target cells. Stromal cells show enhanced proliferation, migration, and secretion of inflammatory cytokines	Transfer of functionally active EV protein and microRNA. miR-150, miR-146a and miR-155 accumulation is increased in target cells comparably to the vesicle accumulation over time. EV proteins were transferred to the target cell membrane after 24 h. This result in activation of transcription factors within the target cells	[137]
Trophoblast derived EVs	Endometrial cells	RNA cargo: intronic- -non-coding region LINC00478, exonic- -coding region LINC00478 and ZNF81	Trophoblast spheroid derived EVs induced drastic transcriptomic alterations mainly related to extracellular matrix remodelling and GPCR mediated signalling in the endometrial cells while the non-trophoblast cell derived EVs failed to induce such changes	Transcripts were transferred from trophoblast cells to endometrial cells and downregulate the endogenous expression of the same transcripts. At least part of the transcriptomic changes could be explained by miRNA content of the trophoblast derived EVs	[78]
EVs from a different cellular source (a human choriocarcinoma cell line JAr and porcine follicular fluid-derived EVs (pFF EVs) and EVs from differently sized bovine follicles (large, medium and small) were incubated with bovine spermatozoa	Bovine spermatozoa	Protein Cargo: Surface protein cargo	Only bovine follicular fluid derived EVs could affect the bovine spermatozoa functional parameters such as sperm viability, capacitation and acrosomal reaction	EVs surface proteins are potentially responsible for the functional effects	[79]
EVs from EV71 infected cells	RD cells	RNA Cargo: viral genomic RNA and miR-146a	EV71 transmission taking place independent of virus-specific receptors	Transfer of miR-146a and functional viral RNA in exosomes to the target cells (EV viral RNA could be transferred to and replicate in a new target cell while the EV miR-146a suppressed type I interferon response in the target cell, thus facilitating the viral replication)	[138]
Mesenchymal stromal cell derived EV	THP-1 monocytes and bone marrow-derived macrophages	RNA cargo: miR-27a-3p	Alleviate lung injury	Directly transferring miR-27a-3p to alveolar macrophages. miR-27a-3p acts to target NFKB1 and regulate M2 macrophage polarization	[139]
Breast cancer tumor cell derived exosomes	Adipose derived stem cells (ASCs)	Protein cargo: transforming growth factor beta (TGF-β)	Recipient cell became more contractile, secreted more vascular endothelial growth factor (VEGF), and promoted angiogenic sprouting of human umbilical vein endothelial cells	These changes were dependent on TGF-β related signaling, and exosomes were found to contain TGF-β that activate MAPK signaling pathways in ASCs	[140]
The exosomes in hepatoma cells	Endothelial cells in tumour stroma	RNA cargo: miR-210	promotes tumour angiogenesis	Direct transfer of miRNA, miR-210 in exosomes targets SMAD 4 and STAT 6 and enhances angiogenesis in endothelial cells. Endogenous miR-210 expression not affected	[141]
Amniotic fluid mesenchymal cell EV	Ovarian cells	RNA cargo: miRNA-21	carrying miRNA-21 play a role in regeneration of ovarian reserve following chemotherapy induced damage	Via transfer of miRNA-21 in exosomes, and modulation of PTEN and caspase 3 apoptotic pathways, that results in cell survival, proliferation and migration	[142]
Metastatic breast cancer cell exosomes	Normal cells in a metastatic environment	RNA cargo: miR-10b	Promote cell invasion and migration in surrounding normal cells	Carrying miR-10b and reduce the protein level of its target genes HOXD10 (homeobox D10) and KLF4 Kruppel-like factor 4	[143]
Colon cancer-cell-derived exosomes (CDEs)	Tumour cells	RNA cargo: ΔNp73 mRNA	Significantly increases the proliferative potential of tumour cells	CDEs is enriched with ΔNp73 mRNA significantly increased the proliferative potential of target cells by inhibiting the function of tumour suppressor gene P53	[144]
Pancreatic adenocarcinoma derived EVs	Endothelial cells	EV surface proteome: tetraspanin: Tspan8	EVs enhanced endothelial cell proliferation, migration, sprouting, and maturation of progenitors	Tspan8 in EVs selectively bind to endothelial cells potentially with the help of other surface molecules (such as CD49d, LamininR1, Mac2BP, and MFGE8) and induce angiogenesis related gene expression	[111]
HEK293F cells	activated human endothelial cells (HMEC-1)	EV surface proteome: integrins: lymphocyte function-associated antigen-1 (LFA-1) receptor and internally loaded peptide (EV^Myd88^)	Reduce the cytokine release by endothelial cells were stimulated first by TNF-α	Increased EV binding to ICAM-1 expressing cells (receptor for LFA-1) and intracellular delivery of Myd88	[145]
human prostate adenocarcinoma cells	tumor necrosis factor-a-activated endothelial cells	EV surface proteome: cell adhesion molecules ICAM-1	blocked leukocyte adhesion to TNF-a-activated endothelial cells	overall avidity of cellular adhesion between immune cells with endothelial cells was regulated affinity and the valency of ICAM-1 in EVs with their receptors on target cell surface	[117]
PC3 human PCa cells	HUVEC cells	EV surface proteome: Integrins α6β1/α6β4 and α2β1 and ganglioside, GM1	induced cell branching morphogenesis in HUVEC cells	Integrin in EVs bind to laminin in target cells facilitated by CD151 and target cell responses are dependent on laminin content on the cell surface	[101]
Human myeloma cells	Myeloma cells, bone marrow stromal cells and human umbilical vein endothelial cells	EV corona: fibronectin	Enhance myeloma tumor growth, progression and umbilical vein endothelial cell invasion	fibronectin-mediated binding of exosomes to myeloma cells activated p38 and pERK signaling	[126]

#### The role of EV molecular cargo in selective EV interaction with recipient cells: RNA and its derivatives

EV are particularly enriched in small, transferable RNAs, including microRNAs (miRNAs), yRNAs, tRNAs, and mRNA fragments. Among these, miRNAs are the most extensively studied and have been shown to mediate intercellular communication by inducing functional and phenotypic changes in recipient cells [146, 147]. Notably, the RNA composition of EVs appears to be cell-type specific. A portion of this RNA cargo is believed to be actively selected and loaded into EVs through mechanisms involving RNA-binding proteins, calcium-binding proteins, or specific sequence motifs, and this loading is influenced by the RNA size and concentration [148]. For instance, miRNAs that are overexpressed in metastatic cancer cells are also more abundant in their secreted EVs compared to those from non-metastatic or normal cells [143, 149]. Sun et al. (2020) demonstrated that both free miR-21 and EV miR-21 elicited similar effects on target gene expression [149]. Other studies provide further evidence of RNA-mediated EV functionality. For example, Es-Haghi et al. (2019) reported the transfer of specific embryonic RNA transcripts to endometrial cells, resulting in altered endogenous transcript levels [150]. Similarly, Godakumara et al. (2021) showed that embryo-derived miRNAs transferred via EVs to endometrial cells silenced their target genes [78]. Squadrito et al. (2014) also found an inverse correlation between miRNA levels in EVs from macrophages and the abundance of their target transcripts in recipient cells, supporting the concept of miRNA-mediated gene silencing [151]. In the context of cancer, EV-associated miRNAs have been implicated in promoting tumourogenesis, invasion, and metastasis [28, 147]. Cancer cell-derived EVs have even been shown to reprogram the proteome of recipient normal cells, transforming them into cells with malignant characteristics and enhancing cancer progression [152]. Interestingly, only EVs derived from cancer cells, but not from normal immune cells, have been shown to stimulate pattern-recognition receptors such as toll-like receptors in peripheral blood mononuclear cells—likely due to immunostimulatory RNA sequences within the EVs [153].

Nevertheless, quantitative studies have revealed that EVs typically contain very low amounts of miRNA—on average, one copy per 121 EVs in seminal fluid and plasma-derived EVs from healthy individuals [154]. These findings have led some researchers to question whether EV-transferred miRNAs can actually exert meaningful biological effects [148, 155]. More research is needed on the mechanisms underlying the release of miRNAs from EVs (endosomal escape) following uptake and their subsequent regulation of gene expression. It is important to note that EVs containing same RNA cargo may elicit different responses depending on the recipient cell type, and only a small fraction of the total transcriptomic changes observed in recipient cells after EV uptake can be directly attributed to EV miRNAs (Godakumara et al., 2021). This indicates that other EV components—such as proteins or lipids—may play a more substantial role in shaping recipient cell responses.

#### The effect of EV molecular cargo on selective EV interaction with recipient cells: Proteins

EVs carry a diverse array of proteins, including both cell type-specific and common proteins, which can be delivered to recipient cells either as membrane-associated or soluble proteins. These protein cargos may include enzymes, transcription factors, and receptors capable of inducing functional and phenotypic alterations in target cells. Proteins sorted in the EVs reflect the parent cells of origin and those proteins are implicated in various functions such as cell adhesion, signal transduction, cytoskeletal organization and membrane trafficking etc. [156]. Multiple mechanisms have been proposed for protein sorting into EVs. The most studied pathway is the Endosomal Sorting Complexes Required for Transport (ESCRT) machinery, which incorporates proteins like tetraspanins and ubiquitin-like proteins (UBLs) involved in post-translational modifications in to EVs [157]. In addition, ESCRT-independent mechanisms also exist, relying on molecules such as neutral sphingomyelinase (nSMase), CD63, syntenin-1, and syndecans [156]. Environmental stimuli can also modulate the EV protein composition. For instance, stress conditions like hypoxia have been shown to elevate levels of heat shock proteins (HSP70, HSP90) within EVs [158, 159]. The targeting ability of EVs is largely attributed to their membrane protein component such as integrins and tetraspanins etc. [160]. In the context of embryo-maternal communication, we previously demonstrated that trophoblast-derived EVs can specifically induce proteomic reprogramming in endometrial cells [161], promoting an implantation-supportive environment. We also identified potential protein cargo molecules responsible for these effects [95], highlighting EV protein transfer as a key mechanism of targeted intercellular communication.

Overall, the proteins carried by EVs, shaped by their cell of origin and physiological state, together with the specific interactions between EV surface proteins and receptors on target cells, likely play a major role in determining how EVs act and what effects they produce. However, the exact mechanisms by which EV protein cargo is delivered to recipient cells and triggers functional changes remain poorly understood. Further studies focusing on specific EV-recipient cell pairs are needed to elucidate the molecular basis of EV tropism and the role of EV protein cargo in mediating targeted cellular responses.

#### The effect of EV molecular cargo in selective EV interaction with recipient cells: Lipids

EVs are enclosed by a lipid bilayer that is highly enriched in membrane lipids such as cholesterol, ceramide, sphingomyelin, and detergent-resistant membrane components, which collectively contribute to their stability in the extracellular environment. Compared to their parent cells, EVs typically contain higher levels of cholesterol, sphingomyelin (SM), glycosphingolipids, and phosphatidylserine [162]. Cholesterol, in particular, plays a critical role in determining the fate of EVs; cholesterol-rich EVs originating from MVBs are more likely to be secreted, whereas those with lower cholesterol content are often directed toward lysosomal degradation [163]. Furthermore, exposure to CD4 + T cell derived EVs has been shown to cause cholesterol accumulation in monocytes and promote the release of proinflammatory cytokines, a process that can be reversed by blocking cell surface receptors such as the phosphatidylserine receptor [164]. The exact mechanisms through which EV and recipient cell lipid composition shapes their biological activity and governs their interactions with specific recipient cell types remain poorly understood and require further investigation.

#### Effects of EV dose in EV selective interaction with recipient cells

The functional impact of EV on recipient cells is dose-dependent [18, 165], with different concentrations eliciting distinct cellular responses [15]. Higher doses consistently supress exocytosis and an increase in lysosomal activity in recipient cells regardless of EV type. In contrast, at lower doses, the effects were more dependent on the cellular origin of EVs, reflecting the functional characteristics of the originating cells [16, 165]. Additionally, non-pigmented ciliary epithelium (NPCE)-derived EVs were shown affect canonical Wnt signaling in a normal trabecular meshwork (NTM) cells in dose dependent manner. These changes differentially affected Pro-MMP9 and MMP9 activity NTM cells [15]. Therefore, EV dose is a critical factor when assessing EV functionality between two cell types. Careful optimization of EV dose is essential to achieve targeted functional effects, even in cell pairs exhibiting EV tropism.

#### Role of time of EV-recipient cell interaction in selective EV interaction with recipient cells

EV uptake generally increases over time and eventually reaches saturation. Substantial EV internalization can occur as early as 5 min post-exposure [70]. However, the release of EV cargo following internalization appears to be a relatively inefficient process. For example, although the proportion of recipient cells internalizing EVs increases over time, less than half of these internalized EVs successfully deliver their cargo [98]. It is important to note that these time-course observations have been made across different EV-recipient cell systems and therefore may not be universally generalizable. EVs are known to trigger both acute and prolonged responses in recipient cells. As exposure time increases, EV uptake typically rises, but EV functionality may also evolve over time. Notably, EV-induced changes in gene expression can be detected as early as 5–30 min after exposure [17], suggesting a rapid cellular response. In contrast, changes in protein expression generally take longer to manifest [15]. Interestingly, EV-induced alterations in the secretory proteins may occur more rapidly than previously anticipated [18]. It is possible EV-mediated receptor-ligand interactions at the cell surface could trigger signaling cascades faster than the processes of endocytosis, cargo release, and downstream signaling. However, this distinction between surface-triggered signaling and cargo-mediated effects is not yet well established. A better understanding of the temporal dynamics of EV uptake stages and their link to specific functional responses would be instrumental in deciphering how EVs exert their biological effects in selected recipient cells.

**Fig. 3:**
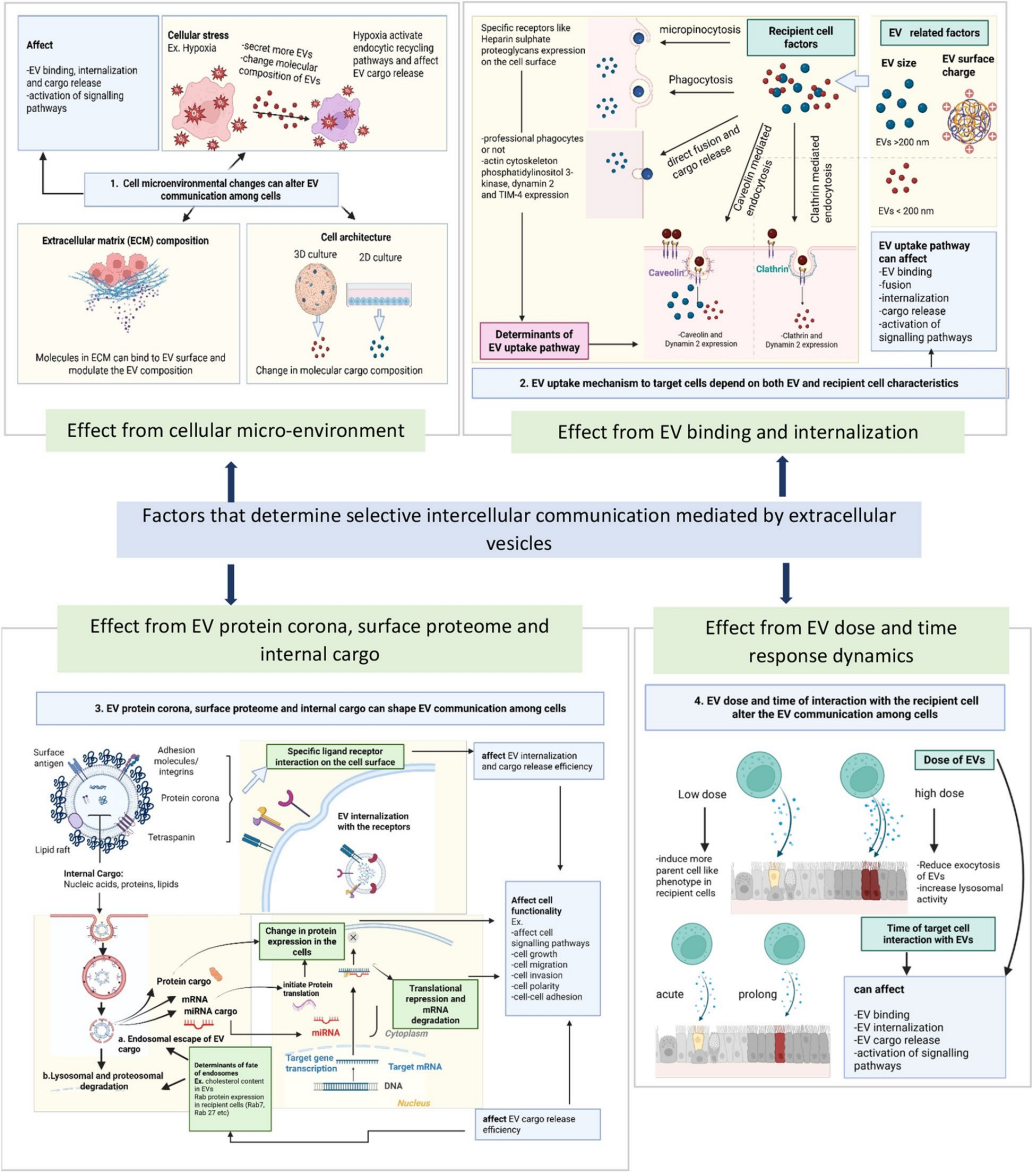
Determinants of specific interactions between extracellular vesicles (EVs) and recipient cells. Multiple factors shape how EVs interact with and influence recipient cells. These include: (1) microenvironmental conditions that modulate EV-cell communication; (2) physicochemical properties of EVs and the uptake pathways activated in recipient cells; (3) EV surface molecules and internal cargo such as proteins, lipids, and RNAs; and (4) EV dose and temporal dynamics. 2D culture: two-dimensional culture; 3D culture: three-dimensional culture; mRNA: messenger RNA; miRNA: microRNA, TIM-4; T-cell immunoglobulin and mucin domain containing 4

In summary, the tuning of EV-mediated cell–cell communication is governed by a complex set of factors, and understanding these determinants is essential for uncovering the mechanisms underlying true EV specificity (Fig. 3).

### Challenges in studying natural EV specificity

*The primary challenge in studying EVs target specificity is the lack of clear and standardized definitions.* In particular, the concept of EV specificity or tropism needs to be more comprehensively defined. Likewise, the term "EV uptake" encompasses several distinct processes—namely, EV binding to the cell surface, membrane fusion, internalization and cargo delivery. Future studies should explicitly specify which level of uptake is being investigated, as this precision is crucial for understanding the mechanisms underlying EV target specificity. Importantly, EV uptake alone is not a reliable endpoint for assessing EV specificity. As supported by previous studies, the presence of selective uptake does not necessarily translate into a corresponding functional response in recipient cells. Therefore, we propose that future investigations should examine the dynamics of EV interactions at both the uptake and functional levels, whenever possible, to better understand patterns of EV specificity and to prioritize functional outcomes as a more relevant endpoint for assessing EV target specificity.

*The second major challenge remains the limited understanding of the link between EV uptake and the resulting functional response.* While EVs can interact with recipient cells through surface binding, membrane fusion, or internalization, the precise mechanisms governing cargo delivery and functional activation are still being elucidated. EV cargo may be released into the cytoplasm of recipient cells via several pathways, including EV direct fusion with the recipient cell membrane, transient “kiss-and-run” fusion with the endoplasmic reticulum, endosomal membrane fusion, or endosomal rupture [108]. Nevertheless, the majority of this cargo appears to be recycled or degraded in lysosomes [166].

Emerging technologies are improving our ability to study EV uptake and cargo release with greater sensitivity and resolution. Among the most promising are CRISPR/Cas9-based guide RNA delivery systems [167] and luminescent-tagged cargo protein assays [98, 168]. These innovative approaches allow researchers to track EV internalization and assess the delivery of bioactive cargo across various cell types in vitro. Findings from these studies indicate that EV uptake and cargo delivery are tightly regulated processes influenced by both EV-intrinsic properties and recipient cell characteristics. However, these studies often overlook the importance of matching between EV type and recipient cell, making it challenging to generalize their findings to systems where EVs exhibit tropism. Therefore, research that compares cargo delivery processes in systems with and without EV tropism would be particularly valuable in this context, as it could provide insights into how EV specificity influences the efficiency and mechanism of cargo delivery. Additionally, mechanistic studies are needed to connect uptake dynamics with EV functionality dynamics, in order to better understand the mechanisms by which EVs drive target specificity.

*Thirdly, we have not yet established appropriate models where EV specificity is clearly defined.* Accurate investigation of EV-cell interactions require careful consideration of both the EV source and the recipient cell type. Experimental systems should be designed using models where EV tropism is either well-characterized or experimentally validated. Moreover, several factors influencing EV function in recipient cells must be accounted for, including molecular cargo composition, EV dose and exposure time, microenvironmental context, EV subtype (e.g., small vs. large EVs), maturation status of the recipient cell, and its cell cycle stage etc. Another often overlooked aspect is the establishment of physiologically relevant EV-to-cell ratios, as excessive EV loading in any system can artificially enhance uptake and compromise biological relevance. Therefore, standardized experimental models using physiologically relevant EV concentrations are essential for accurately elucidating how EVs achieve target specificity and exert biological effects.

*Fourthly,* we encounter different technological limitations in studying EV uptake and function accurately. Fluorescent labelling of EV membranes using lipid dyes, or tagging EV cargo proteins with fluorescent markers, are among the most commonly used methods to visualize and quantify EV internalization. Flow cytometry and confocal microscopy are frequently employed to analyse these labelled EVs [106, 169, 170]. While advanced microscopy techniques can sometimes distinguish between EV binding to the cell surface and actual internalization. flow cytometry lacks this resolution and cannot differentiate between surface-bound and truly internalized EVs. The use of pH-sensitive fluorescent dyes in flow cytometry has partially addressed this limitation by enabling discrimination between surface-associated and internalized EVs to some extent. However, although these approaches detect EV internalization by various cell types, they do not accurately reflect the efficiency or specificity of uptake across different recipient cells. Furthermore, they cannot distinguish between critical steps in the uptake process—such as binding, membrane fusion, and actual cargo delivery. Additionally, lipid-based fluorescent dyes are prone to diffusion into the membranes of recipient cells, often resulting in false-positive signals and overestimation of uptake. Similarly, fluorescent tagging of EV cargo proteins introduces variability depending on the cell type of origin—some cells incorporate fluorescent tags more efficiently than others. As a result, EVs derived from highly tagging-efficient cells may produce stronger signals, while others show weaker signals, regardless of actual uptake levels. This discrepancy can mislead interpretations, giving the false impression of selective uptake, when in reality, the difference stems from the labelling efficiency, not biological tropism. Overall, these limitations highlight the urgent need for more accurate, standardized, and biologically relevant technologies to assess EV internalization and target specificity. In vivo tracking of EV also presents several unique challenges. One major issue is the rapid accumulation of EVs in the mononuclear phagocyte system due to high blood flow in these regions. Additional challenges include the short half-life of EVs and the photobleaching of fluorescent dyes used for labelling over time. Future studies should prioritize the development of more accurate and reliable methods for tracing EV distribution and accumulation in organs. Key variables such as the route of administration, EV dosage, and time-dependent responses must be carefully considered.

Standardization of EV dosing is also crucial. It remains unclear whether treatment doses should be based on total protein content or particle number, such as that determined by single nanoparticle analysis. Likewise, EV isolation methods for such studies need to be standardized to ensure consistency and reproducibility. Time-response studies are equally important, as different EV populations can exert varying effects at different time points. To better understand EV functionality, it is essential to characterize their effects using well-defined dose-response and time-response curves.

### Shifting toward functional readouts of EV activity to assess EV-cell interaction is needed

As noted above, recipient cells may adopt selective mechanisms to internalize EVs. However, it remains unclear whether this selective uptake translates directly into functional selectivity. There is an increasing need to develop and validate assays that assess functional outcomes, including changes in gene expression, protein abundance, or secretion profiles following EV treatment. For example, functional assays that measure the biological effects of EV uptake—such as RT-PCR to detect EV-delivered miRNAs in recipient cells, western blotting/ELISA for assessing changes in protein expression levels—provide more meaningful insights into EV-cell interactions than uptake measurements alone. Integrating these functional assays with EV uptake inhibitors or blockers can help delineate the mechanisms underlying EV-mediated effects, offering a more comprehensive understanding of their biological activity. Such approaches are more biologically relevant than merely tracking EV internalization.

To gain deeper insight into EV tropism, more advanced and precise technologies and models are required to study EV binding, fusion, internalization, cargo delivery and downstream signalling mechanisms. A major goal for future research should be to identify conserved functional responses that can serve as indicators of EV activity across broad range of cell types. This kind of assay has the advantage of studying the natural EV functionality without manipulating cell or the EVs.

## Conclusion

We have given this manuscript the title “Does extracellular vesicle specificity truly exist?”. In the conclusion, we acknowledge that we cannot provide a definitive “yes” or “no” answer. There is overwhelming evidence supporting the idea that EV specificity does exist. However, the underlying mechanisms—how it works, under which conditions it occurs, and what factors regulate it—remain incompletely understood. At the same time, the opposite phenomenon—non-specific interactions of EVs with recipient cells—also appears to be present. Thus, our main conclusion is that both specific and non-specific interactions may coexist. This duality likely reflects the heterogeneity of EV populations, which vary according to the cell type of origin and many other factors that we have discussed in this manuscript.

The question of whether EV specificity exists is a fundamental one, with important implications for the development of EV applications in multiple fields. Yet, it seems to remain a neglected area of EV biology. We hope that this manuscript will stimulate discussion, encourage further investigation, and help bring this important issue to the centre of attention in EV research.

## Data Availability

Data sharing is not applicable to this article as no datasets were generated or analysed during the current study.
